# Facial fluctuating asymmetry is not associated with childhood ill-health in a large British cohort study

**DOI:** 10.1098/rspb.2014.1639

**Published:** 2014-10-07

**Authors:** Nicholas Pound, David W. Lawson, Arshed M. Toma, Stephen Richmond, Alexei I. Zhurov, Ian S. Penton-Voak

**Affiliations:** 1 Division of Psychology, Department of Life Sciences, Brunel University, London UB8 3PH, UK; 2 Department of Population Health, London School of Hygiene and Tropical Medicine, London, UK; 3 Applied Clinical Research and Public Health, Orthodontic Department, School of Dentistry, Cardiff University, Cardiff, UK; 4 School of Experimental Psychology, University of Bristol, Bristol, UK

**Keywords:** ALSPAC, childhood health, developmental stability, facial asymmetry, fluctuating asymmetry, IQ

## Abstract

The idea that symmetry in facial traits is associated with attractiveness because it reliably indicates good physiological health, particularly to potential sexual partners, has generated an extensive literature on the evolution of human mate choice. However, large-scale tests of this hypothesis using direct or longitudinal assessments of physiological health are lacking. Here, we investigate relationships between facial fluctuating asymmetry (FA) and detailed individual health histories in a sample (*n* = 4732) derived from a large longitudinal study (Avon Longitudinal Study of Parents and Children) in South West England. Facial FA was assessed using geometric morphometric analysis of facial landmark configurations derived from three-dimensional facial scans taken at 15 years of age. Facial FA was not associated with longitudinal measures of childhood health. However, there was a very small negative association between facial FA and IQ that remained significant after correcting for a positive allometric relationship between FA and face size. Overall, this study does not support the idea that facial symmetry acts as a reliable cue to physiological health. Consequently, if preferences for facial symmetry do represent an evolved adaptation, then they probably function not to provide marginal fitness benefits by choosing between relatively healthy individuals on the basis of small differences in FA, but rather evolved to motivate avoidance of markers of substantial developmental disturbance and significant pathology.

## 1. Introduction

Fluctuating asymmetry (FA; small random deviations from perfect symmetry in bilateral traits) has been proposed, and is commonly used, as an index of developmental stability: i.e. the ability of an organism to buffer against developmental stressors and perturbations [1,2]. Sources of developmental disturbance may be environmental (e.g. pathogens, toxins, nutritional) but may also be genetic (e.g. mutations), and the accumulation of asymmetries across ontogeny is thought to depend on not just the extent to which an organism is exposed to such perturbations but also its ability to resist them, i.e. developmental stability [3]. Consequently, FA is hypothesized to reflect poor condition, particularly along axes of physiological health [4,5]. Accordingly, measures of FA have been used by researchers as a putative cue to an organism's phenotypic, and possibly underlying genotypic, quality.

In recent years, the use of FA as an index of developmental stability has been popular in evolutionary models of human mate choice and evolutionary psychological studies of sexual preferences, providing a compelling functional explanation for consistent demonstrations that facial symmetry predicts attractiveness in both males and females [6]. This conceptual framework has generated a large literature on preferences for symmetry, and studies of FA in humans fall into two categories: those that assess preferences for symmetry directly (most often studies of faces), and those that assess preferences for traits that may themselves covary with symmetry (such as sexual dimorphism or odour). Although there is much evidence that symmetrical faces are perceived as more attractive (for a review, see [7]), evidence that facial (or indeed bodily) asymmetry is associated with past or present health is equivocal [6]. A recent meta-analysis of the relationship between health, ‘quality’ and asymmetry [8] concluded that for outcome measures across six broad categories (of which one was health and disease specifically while the others included various proxies of quality such as psychological maladaptation and attractiveness) the mean effect size for associations with FA was about *r* = 0.2. There are also indirect indications of publication bias, suggesting the current literature may overestimate the strength of associations between FA and various traits [9] including for reports of relationships between body FA and intelligence [10]. However, a direct attempt to quantify publication bias for FA studies concluded that it was unlikely to be a significant problem [11].

There have been a number of small-scale studies specifically investigating the relationship between health measures and facial asymmetry. Rhodes *et al*. [12] demonstrated that asymmetry assessed from facial landmarks from photographs of 17 year olds born between 1920 and 1929 in the USA did not significantly predict health (determined from medical records) during childhood (*n* = 102) or adolescence (*n* = 192). Similarly, Hume & Montgomerie [13] found that a composite body symmetry score composed of measurements of both facial and other traits was not significantly associated with self-reported health problems in a sample of nearly 200 individuals. Moreover, Honekopp *et al*. [14] reported no significant association between physical fitness and facial asymmetry in 77 young women. Shackelford & Larsen [15] reported inconsistent relationships between facial asymmetry and multiple indices of self-reported health over the previous two months (e.g. runny nose, sore throat, coughs, upset stomach, etc.) across two samples (*n* = 57, *n* = 44) using zero order correlations. In addition, Thornhill & Gangestad [16] found significant relationships between facial asymmetry and self-reported respiratory illness (but not intestinal illness) over the previous 3 years in a sample of around 400 individuals. In general, however, the literature on this topic is characterized by relatively small samples, and for the most part short-term measures of health that are unlikely to capture many relevant aspects of condition or health status during critical periods of development such as prenatal life, infancy and childhood.

Here, we examine possible associations between facial FA and measures of childhood health from a large longitudinal study of British children. Our study design overcomes several common methodological shortcomings of the current literature (notably the comparatively small samples and only short-term self-report measures of health history often employed), enabling an unusually strong test of the functional basis for symmetry preferences in faces. The sample is also more broadly representative of the general population than are samples of university students that have typically been used to investigate correlates of asymmetry. Moreover, included in our analyses are data on birth weight, which is of particular importance since low birth weight (LBW) is associated with morbidity during childhood [17] and it is now becoming apparent that many adult diseases may have their origins during fetal and infant life [18,19]. LBW may arise as a consequence of preterm birth and/or intrauterine growth restriction and there is some evidence that prematurity is associated with developmental instability and consequently, FA [20]. Moreover, recent evidence suggests that prenatal exposure to alcohol alters patterns of directional asymmetry in faces [21].

While concentrating on faces alone is not the strongest test of the developmental instability hypothesis (single traits will have a noisy relationship with developmental instability [3]), the very large representative sample of young people in South West England [22] that we employ, coupled with the excellent longitudinal health history data and high-quality three-dimensional scans of the faces from this cohort go some way to ameliorate this concern and provide for a robust test of associations between health and facial FA.

## 2. Material and methods

All data were sourced from the Avon Longitudinal Study of Parents and Children (ALSPAC), an on-going cohort study initially involving over 14 000 British families with children born in 1991/1992 and with approximately 5500 children participating in data collection aged 15–16 [22]. All data collection was approved by the ALSPAC Ethics and Law Committee, University of Bristol and the Local Research Ethics Committees. Please note that the study website contains details of all the data that are available through a fully searchable data dictionary (http://www.bris.ac.uk/alspac/researchers/data-access/data-dictionary/).

### (a) Geometric morphometric analysis of face shape and size

Analyses reported here are based on 4732 children (2226 males and 2506 females) from the ALSPAC cohort recruited for facial scanning by Toma *et al*. [23], at a mean age of 15.4 years (±0.28 years). Approximately 5500 children participated in ALSPAC data collection (i.e. completed postal questionnaires or clinic assessments) aged 15–16 [22]. Consequently, the sample here includes the majority of ongoing active participants in ALSPAC at that age. For the participants who volunteered, three-dimensional facial images were captured using two high-resolution Konica/Minolta laser scanners following the procedure described previously [23,24]. Twenty-one facial landmarks (electronic supplementary material, figure S1) defined by Farkas [25] were then manually delineated on the three-dimensional facial shells and the X, Y and Z coordinates recorded according to procedures detailed in Toma *et al*. [26]. Measurement precision was examined using the sample of 30 children (15 males and 15 females) from Toma *et al*. [26] for which the landmarks were delineated on each three-dimensional facial shell separately by two independent examiners.

Geometric morphometric analyses were carried out to examine variations in face shape and symmetry using the MorphoJ software package [27]. Procrustes registration was first used to remove scale, rotational and translational differences so that shape variation could be isolated [28]. For symmetric objects, such as the three-dimensional face shells in this study, a specialized approach is taken that separates the symmetric and asymmetric components of shape variation (for details, see [29]). Measurement error associated with the landmark delineation process was quantified using Procrustes ANOVA [29] for the sample of faces with replicate measures.

Following this, for the main sample (*n* = 4732) facial FA was measured using methods similar to those used in previous recent studies on facial FA [30–32]. The Procrustes ANOVA procedure in MorphoJ [27] was used to calculate individual FA scores which correspond to the difference in shape between the left and right sides of the face after correction for directional asymmetry (i.e. after subtracting the mean shape asymmetry for the sample). This method generates two measures of asymmetry, (i) a measure of absolute asymmetry based on Procrustes distances that treats all aspects of shape variation equally and (ii) a measure of the relative magnitude of asymmetries based on Mahalanobis distances that assesses variation relative to variability in the sample, with asymmetry in shape features that are relatively invariant being weighted more heavily [33]. In addition, the Procrustes superimposition process yields a measure of the size of the landmark configurations, namely centroid size [28].

### (b) Health, socioeconomic and demographic data

Three key longitudinal measures of childhood illness were derived from annual postal questionnaires completed by each child's primary carer: ‘Proportion Years Unwell’, ‘Average Symptoms Per Year’ and ‘Total Infection Load’. On 12 questionnaires, administered at 18, 30, 42, 57, 69, 81, 91, 103, 128, 140, 157 and 166 months of age (i.e. during the child's 2nd to 14th years), carers were asked to indicate whether or not the child had experienced any ‘health problems’ during the previous 12 months. We calculated ‘Proportion Years Unwell’ as the proportion of years with valid responses for which ‘health problems’ were reported. Some responses were available for 4688 cases and responses were complete (valid responses for all 12 years) in 2006 cases (42.4%). The mean proportion of years with health problems was slightly lower (*t* = 3.43, d.f. = 4686, *p* < 0.001) for cases with complete data (42.3%) than for those with incomplete data (45.4%). Consequently, to address this possible source of bias we also carried out analyses for a larger, more inclusive, sample of the 4189 (88.5%) cases with mostly complete (6 or more years valid) data on years unwell. For this larger sample, the mean proportion of years with health problems (43.8%) did not differ significantly from the mean for those (*n* = 499) with at least 1 but fewer than 6 years of valid data (46.4%).

On eight questionnaires administered at 6, 18, 30, 42, 81, 91, 103 and 128 months of age (i.e. during the child's 1st to 11th years) carers were asked to report whether or not 10 specific symptoms of illness had been exhibited by the child during the previous 12 months (diarrhoea, vomiting, cough, high temperature, cold, earache, colic or stomach ache, rash, wheezing, breathlessness). The 57 and 69 month questionnaires were not included since their symptom questions referred to time periods more than 12 months that overlapped with other questionnaire periods. So for each year a child could have a symptom score from 0 to 10. We calculated ‘Average Symptoms Per Year’ as the average number of symptoms per year for those years with valid responses. Some responses were available for 4656 cases and responses were complete (valid responses for all 8 years) in 2710 cases (57.3%). The sample with complete data reported fewer (*t* = 2.89, d.f. = 4654, *p* < 0.01) symptoms per year on average (4.26 ± 1.19) than the cases with incomplete data (4.37 ± 1.37). Consequently, as above we also carried out analyses for a larger, more inclusive, sample of the 4270 (90.2%) cases with mostly complete data (5 or more years with valid responses).

‘Total Infection Load’ was calculated as the total number of a list of 16 infections (measles, chicken pox, mumps, meningitis, cold sores, whooping cough, urinary infection, eye infection, chest infection, tonsillitis or laryngitis, German measles, scarlet fever, influenza, cold, glandular fever) the child is reported to have ever experienced based on a single questionnaire at 157 months of age (i.e. aged 13). Scores could range from 0 to 16. On this retrospective infection questionnaire, all items were complete for 3758 children.

In addition, we also considered other possible markers of healthy development. These were birth weight and measures taken in study year 10 (height, weight and BMI measured by ALSPAC researchers). At the point of data collection for study year 10, child ages ranged from 118 to 147 months of age (mean 127.4 ± 2.8). Although 95.3% were between 120 and 132 months of age to account for this variability, when examining associations between growth measures and FA, partial correlations controlling for age have been used. Finally, as an additional developmental outcome we included the results of an IQ test carried out at age 8 (age-adjusted shortened form of the Wechsler Intelligence Scale for Children, 3rd Revision, Psychological Corporation, London, UK).

## 3. Results

### (a) Landmark reproducibility

The intra- and inter-examiner reproducibility of the landmarks used here was examined in a previous study [26], in which for a sample of 30 children (15 males and 15 females) the landmarks were delineated on each three-dimensional facial shell by two independent examiners. They found the majority of X–Y–Z coordinates were reproducible to within less than 1 mm. To further examine the reproducibility of the coordinates, we have computed intraclass correlation coefficients [34] for each landmark's coordinates for each axis as delineated by two examiners in Toma *et al*. [26] (see the electronic supplementary material, table S1 for these analyses). Single measure intraclass correlation coefficients (2,1) are reported since the objective was to establish the reliability of measurements obtained from a single examiner for a larger sample. Reproducibility was good for most landmarks in all three axes (ICC > 0.90 for 41/63; ICC > 0.80 for 60/63 X, Y and Z coordinates).

Additionally, for the sample of 30 faces for which duplicate measurements by independent examiners were available we carried out a Procrustes ANOVA [35] to estimate the amount of measurement error for shape associated with the landmark delineation process (electronic supplementary material, table S2). The mean squares for individual variation, directional asymmetry and FA were 30.4, 91.8 and 3.2 times greater than the measurement error component, respectively. This indicates that measurement error was negligible relative to most of the biological variation being assessed here (e.g. directional asymmetry). However, it may be non-negligible in relation to FA (within facial landmarks) assessed in this way, so results need to be treated with some caution since measurement error could mask small associations between Procrustes FA scores and health/development variables. Single measure intraclass correlation coefficients (2,1) indicated that repeatability was reasonably good for the Procrustes FA scores (0.77, 95% CI [0.58, 0.89]) but poor for the scores based on Mahalanobis distances (0.08, 95% CI [−0.28, 0.42]). Consequently, the Procrustes FA scores were used in subsequent analyses.

### (b) Asymmetry analysis

The Procrustes ANOVA for the main sample yielded FA scores—i.e. the individual asymmetries of shape deviations from the mean asymmetry—that were found to be positively associated with centroid size (*r* = –0.064, *n* = 4732, *p* < 0.0001). Consequently, to control for this positive allometric relationship we also report results using residuals from the FA score—centroid size regression as a measure of asymmetry corrected for centroid size. This is of particular importance for some measures since centroid size itself, an index of face (and therefore head) size, was significantly positively associated with other variables. In particular, it was correlated with birth weight (*r* = 0.196, *n* = 4450, *p* < 0.0001) and also there were significant partial correlations between centroid size and height (*r* = 0.336, d.f. = 4372, *p* < 0.0001), weight (*r* = 0.237, d.f. = 4386, *p* < 0.0001) and BMI (*r* = 0.131, d.f. = 4364, *p* < 0.0001) in year 10 (controlling for age at the time of measurement).

### (c) Associations with childhood health and development

The sample for whom face scan data were available (*n* = 4732) did not differ substantially from the ALSPAC sample as a whole in terms of their history of the exposure to the 16 specific infections used to calculate the Total Infection Load score (see the electronic supplementary material, table S3). Compared to the full ALSPAC cohort, attendees at age 15 did have slightly higher average birth weight and birth length [36]. However, these differences were extremely small (0.5% and 0.2% greater, respectively) and the median number of symptoms of illness reported during each of the first four waves of data collection (4, 5, 5 and 5, respectively) for this sample (*n* = 4732) was identical to the median numbers reported by Hay *et al.* [37] for the larger sample of children with complete symptom data for that period (*n* = 7727).

To examine the associations between measures of childhood health and the asymmetry scores, Pearson's correlation coefficients were calculated. There were no significant associations between any of the composite health measures (Total Infection Load, Proportion Years Unwell, Average Symptoms Per Year) and FA scores (table 1). Moreover, for 15 of the 16 individual infectious diseases that contributed to the Total Infection Load score, there were no significant differences (all *t* < 1.6, *p* > 0.1) in FA between children who had and those who had not experienced the condition by age 13 (see the electronic supplementary material, table S4). FA scores were greater for children who had experienced an ear infection (*t* = 2.28, d.f. = 3973, *p* = 0.023). However, this difference was no longer significant after a Bonferroni correction for multiple comparisons (*n* = 16). Also there were no significant partial correlations between height, weight or BMI in year 10 and FA (controlling for age). However, there was a small significant association between Procrustes FA scores and IQ at age 8 (*r* = –0.044, *n* = 4153, *p* < 0.01) that remained significant controlling for centroid size (*r* = −0.037, *n* = 4153, *p* < 0.05).

**Table 1.** Associations between health/developmental variables, FA scores and centroid size in the full sample and low birth weight sub-sample.

	correlation with						
		Procrustes FA scores		centroid size		FA scores (controlling for centroid size)	
health/developmental variable	*n*	*r*	*p*-value	*r*	*p*-value	*r*	*p*-value
**full sample (*n* = 4732)**							
total infection load (number of infections)	3758	−0.021	0.200	−0.019	0.236	−0.022	0.174
proportion years unwell (complete data)	2006	0.002	0.929	0.019	0.401	0.003	0.886
proportion years unwell (6 or more years data complete)	4189	0.009	0.563	0.001	0.966	0.009	0.560
average symptoms per year (complete data)	2710	−0.009	0.638	0.032	0.097	−0.007	0.715
average symptoms per year (5 or more years data complete)	4270	−0.003	0.845	0.024	0.124	−0.001	0.922
IQ age 8	4153	−0.044**	0.005	0.098***	<0.0001	−0.037*	0.016
birth weight (g)	4450	−0.031*	0.039	0.196***	<0.0001	−0.018	0.219
height (mm) in year 10^a^	4375	−0.013	0.388	0.336***	<0.0001	0.008	0.575
weight (g) in year 10^a^	4389	−0.029	0.055	0.237***	<0.0001	−0.014	0.358
BMI in year 10^a^	4367	−0.030	0.049	0.131***	<0.0001	−0.021	0.156
**low (<2500 g) birth weight sample (*n* = 227)**							
total infection load (number of infections)	173	−0.109	0.154	−0.017	0.822	−0.110	0.148
proportion years unwell (complete data)	87	−0.144	0.183	0.090	0.409	−0.138	0.201
proportion years unwell (6 or more years data complete)	200	0.004	0.952	0.026	0.710	0.006	0.934
average symptoms per year (complete data)	126	−0.017	0.852	0.105	0.241	−0.010	0.911
average symptoms per year (5 or more years data complete)	208	−0.049	0.485	0.011	0.871	−0.048	0.492
IQ age 8	198	−0.166*	0.020	0.098	0.169	−0.159*	0.025
height (mm) in year 10^a^	209	−0.108	0.122	0.453***	<0.0001	−0.079	0.256
weight (g) in year 10^a^	211	−0.052	0.452	0.296***	<0.0001	−0.034	0.625
BMI in year 10^a^	208	0.028	0.688	0.126	0.070	0.036	0.608

*p < 0.05, **p < 0.01, ***p < 0.001.

^a^Partial correlation controlling for age at time of measurement.

There was a small negative association between FA scores and birth weight (*r* =−0.031, *n* = 4450, *p* < 0.05), which was no longer significant after controlling for centroid size. However, in the light of this, and the existence of a more substantial association between birth weight and centroid size (*r* = 0.196, *n* = 4450, *p* < 0.0001), exploratory analyses were conducted to determine whether there might be associations between any of the health variables and asymmetry in a sub-sample of individuals who experienced significant health problems during very early development, i.e. those with low (less than 2500 g) birth weight [38]. Birth weight was available in 4450 cases and was less than 2500 g for 227 individuals (119 males and 108 females). Within this sample, there were also no significant associations between any of the health measures (Total Infection Load, Proportion Years Unwell, Average Symptoms Per Year) and FA scores (table 1). By contrast, the negative association between Procrustes FA scores and IQ at age 8 was stronger in this sub-sample (*r* = −0.166, *n* = 198, *p* < 0.05) and also remained significant after controlling for centroid size (*r* = −0.159, *n* = 198, *p* < 0.05). Moreover, there were no significant partial correlations between FA and height, weight or BMI in year 10 (controlling for age). Follow-up analyses revealed that the negative association between FA scores and IQ was only significant for males (*r* = −0.056, *n* = 1955, *p* < 0.05) but not females (*r* = −0.032, *n* = 2198, *p* = 0.130) with the same association being seen in males (*r* = –0.051, *n* = 1955, *p* < 0.05) but not females (*r* = −0.028, *n* = 2198, *p* = 0.190) after controlling for centroid size.

## 4. Discussion

The large and representative nature of our sample, and high-quality, longitudinal measures of health included make this study one of the strongest tests of the hypothesized association between facial FA and health so far conducted. In this sample, we found no evidence of associations between facial FA and longitudinal health measures, which suggests that although gross facial asymmetries may be associated with specific pathological processes and injuries, subtle variations in facial symmetry (i.e. FA) are not associated with variations in general health during childhood. However, we did find a small significant negative association between facial FA and IQ at age 8 in males that remained significant after controlling for centroid size, which is consistent with the idea that low FA is associated with improved developmental outcomes. The magnitude of this association between facial FA and IQ was somewhat smaller than the estimates of the population correlation between body FA and intelligence (in the range −0.12 to −0.20) reported by a previous meta-analysis [10]. The sample size in this study is much larger (more than 15×) than any of those included in that meta-analysis but it is noteworthy that the effect size reported here is very similar to those found in the largest (*n* > 200) of the previous published studies reporting negative associations between FA and intelligence: −0.07 [39] and −0.13 [40].

The possibility of a positive allometric relationship between facial FA and face size (centroid size), of the type identified here, should be taken into account by researchers investigating associations between facial FA and a range of developmental outcomes. This is particularly important given the non-trivial association identified between birth weight, known to be associated with childhood health outcomes [17], and face (centroid) size during adolescence. Without controls for allometry, factors that increase overall, and/or face, size (e.g. improved nutrition, endocrine processes) could obscure associations between developmental outcomes and FA.

Notwithstanding the small association between FA and IQ, the general lack of associations between facial asymmetry and longitudinal health measures suggests that preferences for symmetrical faces are unlikely to be explained via incurred adaptive benefits of choosing mates of high phenotypic quality. Some caution is needed in interpreting this pattern of findings given that the data are derived from the socioecological context of a modern Western population. However, there are other mechanisms that could plausibly have led to the evolution of facial symmetry preferences. For example, it has been argued that symmetry preferences may arise as a non-functional by-product of cognitive recognition processes, as the arithmetic mean of traits showing FA is zero asymmetry [41–43]. This perceptual bias explanation, however, is not consistent with the finding of greater symmetry preferences for upright than for inverted faces [44]. Moreover, the finding of a small negative association between FA and IQ suggests that facial FA does have the potential to signal some useful information. But given that the association accounts for less than 1% of the observed variation in IQ its real-world importance is questionable, particularly given the availability of more direct cues to intelligence. Nevertheless, further research investigating the developmental processes by which such an association may arise could help shed light on the potential signalling value of FA.

We suggest that if preferences for symmetry do represent an evolved adaptation, then it is not likely that the function is to provide marginal fitness benefits by choosing between relatively healthy individuals. Although small variations in asymmetry between largely healthy individuals may be functionally irrelevant in terms of signalling health, or cueing ‘good genes’, it remains the case that various genetic disorders [5,45] and pathological processes or trauma early in development result in large and easily visible anatomical asymmetries (for reviews, see [5,46–48]). Consequently, preference for the absence of subtle asymmetry could reflect an overgeneralization from an aversion to gross asymmetries [49]. So a preference for symmetry could potentially be maintained if it evolved to motivate avoidance of markers of substantial developmental disturbance and significant pathology. This argument is related to, but subtly different from, others that have suggested gains in fitness as a result of favouring symmetrical mates in the normal range of asymmetry. Overgeneralization effects are common in social perception (e.g. attributing childlike personality traits to ‘babyfaced’ adults [50]). In the case of facial symmetry, the cost–benefit properties of facial stimuli may favour a preference for symmetry even in cases where it is effectively information free (i.e. in the ‘normal’ range) due to the potential costs associated with picking a mate with a serious developmental problem.

A detailed analysis of the ALSPAC cohort revealed that the demographic profile of the recruitment area, and the effects of non-random attrition, have led more affluent groups to be over-represented, and non-White ethnic minority groups to be under-represented relative to the national population [22]. Nevertheless, with a large general population sample, high-quality data on child health and repeated data collection spanning over a decade, the ALSPAC dataset offers substantial advantages over the relatively small studies that have previously been used to test for associations between ill-health and asymmetry. It is possible that the failure to find associations between health and facial FA in this study and others could be due to modern medicine limiting the magnitude of environmental sources of developmental disturbance (e.g. with treatments for pathogens and reduced nutritional stress). On the other hand, in modern populations, the accumulation over recent generations of mildly deleterious mutations that do not significantly impair survival to reproductive age, or fertility, may render contemporary children more, not less, vulnerable to certain sources of developmental stress [51]. Furthermore, previous studies have demonstrated substantial socioeconomic gradients in health (particularly in the biomarkers) in modern populations (e.g. [52,53]) and the relatively wealthy and well-nourished conditions of modern Western society do not fully buffer children against the health costs associated with variable quality rearing environments (e.g. [54]). To clarify this issue, future research will require measures of FA and longitudinal data on child health in non-Western populations with levels of developmental stress more characteristic of our evolutionary past.

## Data accessibility

All the data in this study were derived from the ALSPAC. The ALSPAC study website contains details of all the data that are available through a fully searchable data dictionary (www.bris.ac.uk/alspac/researchers/data-access/data-dictionary/).
